# Clinical Characteristics of Strongyloidiasis during the COVID-19 Pandemic: Systematic Scoping Review

**DOI:** 10.4269/ajtmh.22-0671

**Published:** 2023-03-20

**Authors:** Manasawee Tanariyakul, Bolin Chang, Koichi Keitoku, Marissa Su, Hideharu Hagiya, Yoshito Nishimura

**Affiliations:** 1 Department of Medicine, John A. Burns School of Medicine, University of Hawai’i, Honolulu, Hawaii;; 2 Department of General Medicine, Okayama University Graduate School of Medicine, Dentistry and Pharmaceutical Sciences, Okayama, Japan

## Abstract

The clinical impact of *Strongyloides stercoralis* hyperinfection secondary to immunosuppressive therapy for coronavirus disease 2019 (COVID-19) has been an emerging topic of interest, although characteristics of *Strongyloides* infection in COVID-19 patients are not yet well characterized. This study summarizes the existing evidence of *Strongyloides* infection in COVID-19 patients and recommends future areas of research. According to the PRISMA Extension for Scoping Reviews, we performed a search on MEDLINE and EMBASE for articles with keywords including “*Strongyloides*,” “Strongyloidiasis,” and “COVID-19” from the inception of these databases to June 5, 2022. A total of 104 articles were found. After excluding duplication and thorough reviews, 11 articles, including two observational studies, one conference abstract, and nine case reports or series, were included. Two observational studies focused on revealing the prevalence of *Strongyloides* screening in COVID-19 patients and clinical follow-up. Among the included cases, patients were mostly from low- or middle-income countries and suffered from severe or critical COVID-19. *Strongyloides* hyperinfection and disseminated infection were reported in 60% and 20%, respectively. Interestingly, 40% did not have eosinophilia, a hallmark of parasitic infection, potentially leading to delay in diagnosis of strongyloidiasis. This systematic review summarizes the clinical characteristics of strongyloidiasis in COVID-19 infection. Although further studies to identify risks and precipitants associated with the onset of strongyloidiasis are crucial, increased awareness of the critical condition is warranted.

## INTRODUCTION

The coronavirus disease 2019 (COVID-19) pandemic has significantly affected the global public health landscape.^1^ Immunosuppressive agents have been suggested to alleviate the hyperinflammatory process involved in the pathogenesis of severe COVID-19. Since the publication of the RECOVERY trial and REMAP-CAP trial,^2^^,^^3^ glucocorticoid and anti–interleukin-6 antibodies have been widely used worldwide. However, these medications can reactivate or worsen underlying indolent infections such as tuberculosis, cytomegalovirus, and strongyloidiasis.

Strongyloidiasis is an infection caused by *Strongyloides stercoralis*, a globally prevalent nematode. Human infection occurs transcutaneously by contact with infectious filariform larvae in contaminated soil. Filariform larvae enter the circulation, migrate to the lungs, ascend the tracheobronchial tree, and reach the small intestine after swallowing. In the intestine, the larvae become adult, and then parthenogenetic adult females lay eggs in the intestinal mucosa. The eggs hatch into noninfectious rhabditiform larvae that are shed in the stool. A distinctive feature of *S. stercoralis* is autoinfection in which rhabditiform larvae develop into filariform larvae in the intestine and penetrate colonic mucosa or perianal skin, leading to persistent infection over years and sometimes decades.^4^ Other than autoinfection, rhabditiform larvae can develop and become filariform larvae and infect human through penetration of skin. Rhabditiform larvae can also grow into adult worms and produce eggs that eventually become rhabditiform larvae.

Global prevalence estimates are challenging, mainly due to a lack of recognition and limited statistics. Still, most recent data showed that up to 40% of the population in many tropical or subtropical countries are latently infected with the parasite. In developed countries with a lower prevalence, certain people, such as immigrants, are at high risk for this infection.^5^^,^^6^ Strongyloidiasis may cause mild gastrointestinal or respiratory symptoms, or, with larva currens, a rapidly moving pruritic linear skin eruption, but many cases are asymptomatic. However, if the immune system is compromised by corticosteroid use or human T-cell leukemia/lymphoma virus type 1 infection,^7^ uncontrolled proliferation and dissemination of the parasite, known as *Strongyloides stercoralis* hyperinfection syndrome (SHS), occur. Although infection is limited to the gastrointestinal tract and lungs in SHS, the larvae are spread to all organs in disseminated strongyloidiasis. If untreated, the mortality rate can approach 100%.^4^ Even before the COVID-19 pandemic, with the advent of transplant and immunosuppressive therapy, SHS was an emergent health care issue as one of the neglected tropical diseases.^8^ Since the pandemic, several cases of SHS have been reported in patients with COVID-19, likely due to the use of immunosuppressive agents, further unearthing the hidden burden of strongyloidiasis.^9^ Both SHS and COVID-19 patients manifest septic shock, respiratory failure, secondary bacterial infection, or bilateral lung infiltrates; therefore, diagnosis of SHS is challenging in the setting of COVID-19 pandemic.

Although strongyloidiasis is critical yet curable, there is no systematic review regarding the association between *S. stercoralis* infection and COVID-19. Thus, we performed a scoping review to identify its clinical characteristics, diagnostics method, and treatment.

## MATERIALS AND METHODS

### Study design.

We performed a systematic scoping review in accordance with the Preferred Reporting Items for Systematic Reviews and Meta-Analyses (PRISMA) extension for scoping reviews (PRISMA-ScR).^10^^,^^11^ Supplemental Appendix 1 is the PRISMA-ScR checklist of the present study.

### Search strategy.

MEDLINE and EMBASE searches were conducted for all peer-reviewed articles from inception to June 5, 2022. We used no filters for study design and language. Additional relevant articles were screened with the reference lists of all articles that satisfied the eligibility criteria. The search strategy harbored relevant keywords, including “*Strongyloides*,” “Strongyloidiasis,” and “COVID-19.” Two authors (M. S. and Y. N.) searched independently. See Supplemental Appendix 2 for detailed search terms.

### Eligibility criteria.

The criteria for the inclusion of articles were 1) articles describing the relationship between strongyloidiasis and COVID-19 or cases of strongyloidiasis in patients with laboratory-confirmed COVID-19, including conference abstracts, and 2) randomized controlled trials, case-control studies, cohort studies (prospective or retrospective), cross-sectional studies, and case series. The exclusion criteria included 1) qualitative studies, review articles, and commentaries and 2) diagnosis of COVID-19 made without confirmatory polymerase chain reaction testing.

### Study selection.

Two authors (M. S. and Y. N.) assessed selected articles for full-text assessment independently using EndNote 20 reference management software. Articles considered eligible were subsequently evaluated in full length.

### Data extraction and definition.

We used a standardized data collection form that followed the PRISMA and Cochrane Collaboration guidelines for systematic reviews to obtain the following information from each study: title, name of authors, year of publication, country of origin, study characteristics, target outcome, aims, study and comparative groups, key findings, and limitations. We also summarized data from included cases to identify clinical characteristics of strongyloidiasis in COVID-19.

## RESULTS

### Search results and study selection.

**Figure 1.**
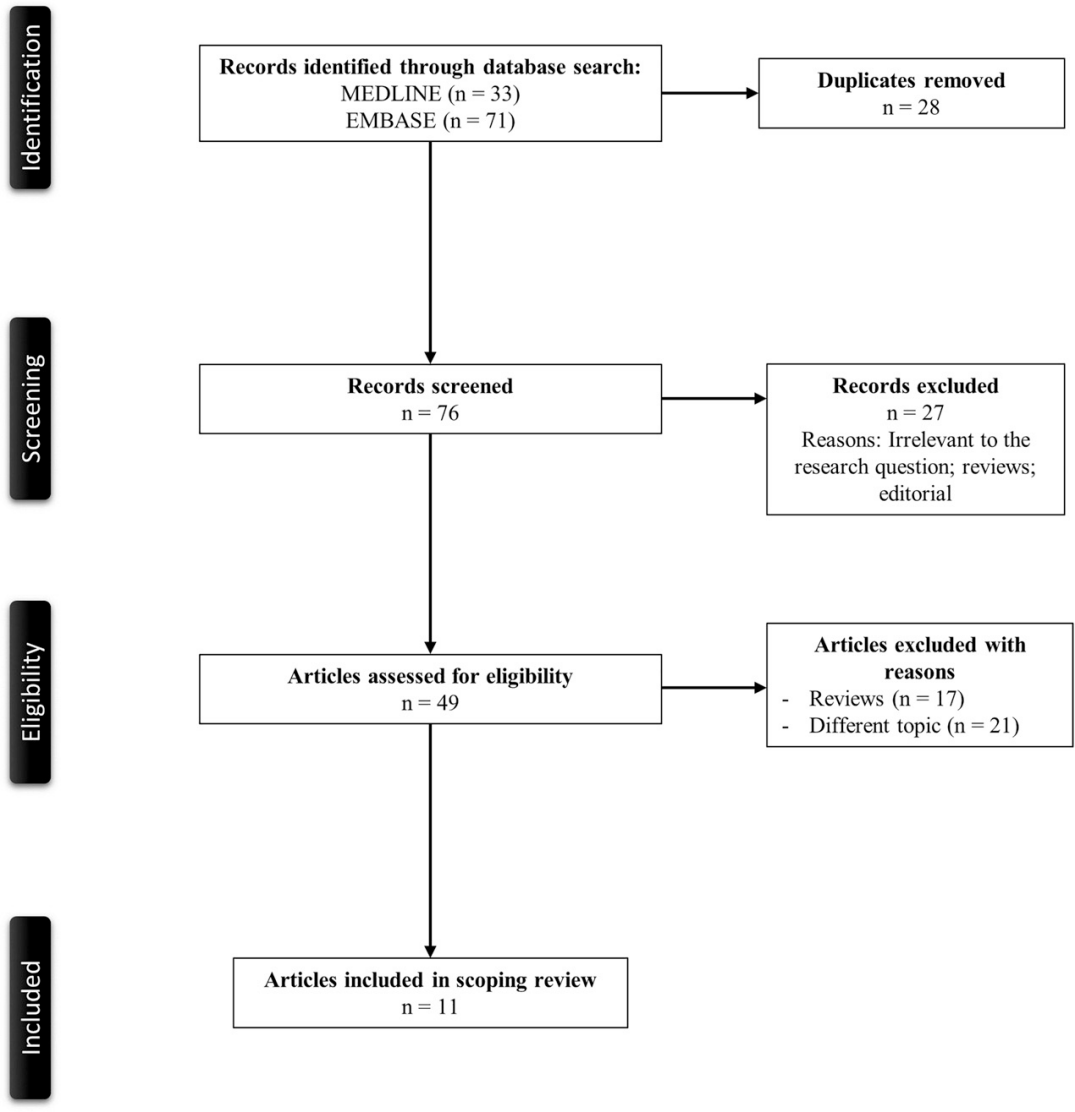
PRISMA flowchart of the search strategy.

Figure 1 illustrates a PRISMA flow diagram that depicts the process of identification, screening, eligibility, and inclusion or exclusion of the studies. The initial search of MEDLINE and EMBASE databases yielded 33 and 71 articles, respectively. Twenty-eight duplicate studies were removed. Seventy-six articles were screened based on their relevance and type of article. Twenty-seven articles that were either review articles or editorials were excluded from the study. Forty-nine articles were then evaluated for full-text review per our eligibility criteria for study inclusion. Thirty-eight reviews, opinion articles, and articles with irrelevant topics were excluded. A total of 11 articles with two observational studies and nine case reports or series were included in the review.

### Description of included studies.

Table 1 describes the main characteristics of two observational studies from the scoping review.^12^^,^^13^ Overall, these two studies were observational studies without comparative groups to evaluate the characteristics of strongyloidiasis in COVID-19, specifically the effect of drugs used in COVID-19 on the evolution of strongyloidiasis and the current situation regarding diagnostic and treatment protocols for strongyloidiasis in COVID-19 patients.

**Table 1:** Main characteristics of the included observational studies in the scoping review

Study (country)	Study type	Aim	Outcome	Population	Comparative groups	Key findings	Limitations
Lorenzo et al., 2022 (Spain)	Longitudinal	To evaluate the frequency of strongyloidiasis in immigrants with COVID-19 and the clinical characteristics of these patients	Investigational	Patients > 18 years from strongyloidiasis endemic areas with COVID-19 (*N* = 86)	N/A	The use of dexamethasone in patients with COVID-19 did not affect the clinical course or evolution of strongyloidiasis; new strongyloidiasis is often paucisymptomatic or asymptomatic during COVID-19 infections	Small sample size with lack of generalizability; patients with a previous diagnosis of strongyloidiasis were excluded; cases with nonserological materials were excluded
Rodríguez-Guardado et al., 2022 (Spain)	Cross-sectional	To glean a better understanding of the diagnostic and therapeutic situation of strongyloidiasis in Spain and more specifically in patients coinfected with COVID-19	Investigational	121 responses from SEIMC members during February and March 2021	N/A	Most centers surveyed (84, 69.5%) had no specific strongyloidiasis screening protocol, and only 22 centers (18%) screened strongyloidiasis in patients with COVID-19	Survey-based design caused selection bias; the real prevalence was not estimated because participants were not asked about the number of cases screened; limited data on the characteristics of patients with hyperinfestation

COVID-19 = coronavirus disease 2019; SEIMC = Spanish Society of Infectious Diseases and Clinical Microbiology.

Lorenzo et al.^12^ conducted a longitudinal study by reviewing patients diagnosed with COVID-19 from a tertiary care hospital in central-western Spain. Although their sample size was small (*N* = 86), they identified seven patients with strongyloidiasis; of the seven, three received dexamethasone, and none received tocilizumab. The authors found that the use of dexamethasone in patients with COVID-19 and strongyloidiasis did not affect the clinical course of strongyloidiasis, and new strongyloidiasis infections were often pauci-symptomatic or asymptomatic. A cross-sectional survey by Rodríguez-Guardado et al.,^13^ which included responses from 121 different centers from the Spanish Society of Infectious Diseases and Clinical Microbiology, identified 227 cases of strongyloidiasis in patients with COVID-19, where four patients developed massive hyperinfestation syndrome, leading to one death. However, the authors noted that most centers surveyed (*N* = 84, 69.5%) had no specific strongyloidiasis screening protocol, and only 22 centers (18.2%) screened for strongyloidiasis in patients with COVID-19. Among the 22 centers, 16 conducted only serological testing for *Strongyloides* screening.

### Review of included cases.

Table 2 delineates the demographics and clinical characteristics of patients with *Strongyloides* infection and COVID-19 (*N* = 10),^9^^,^^14^^–^^21^ with a predominance of female patients (30% male and 70% female). Fifty percent presented with critical COVID-19, and 40% had severe COVID-19. Among the cases, 50% were admitted to the ICU, but no deaths were reported. Initial symptoms were predominantly fever (40%) followed by cough (30%), pruritus (20%), and rash (10%). SHS was the predominant infection status (60%), with most cases diagnosed by stool testing (70%). *Strongyloides* infection was often associated with leukocytosis (60%) and eosinophilia (60%). Every patient received immunosuppressive therapy. Seventy percent received a prednisone equivalent of at least 40 mg/day, 10% received a prolonged corticosteroid treatment of > 2 weeks, and 10% received no corticosteroid treatment. Thirty percent received additional biologic therapy. Regardless of *Strongyloides* infection status, ivermectin was the predominant treatment used for *Strongyloides*, and 20% received both ivermectin and albendazole.

**Table 2:** Baseline demographics, laboratory findings, and chief features of the patients from case reports and case series

Demographic	Prevalence* (%)	Median (IQR)
Age, years	–	55.5 (44.8–62.3)
Sex		
Male	3/10 (30.0)	–
Female	7/10 (70.0)	–
Country of origin		
Ecuador	3/10	–
Bolivia	1/10	–
India	1/10	–
Italy	1/10	–
Peru	1/10	–
Spain	1/10	–
Nicaragua	1/10	–
Honduras	1/10	–
Initial symptoms		
Fever	4/10 (40.0)	–
Cough	3/10 (30.0)	–
Pruritus	2/10 (20.0)	–
Rash	1/10 (10.0)	–
COVID-19 severity		
Mild	1/10 (10.0)	–
Severe	4/10 (40.0)	–
Critical illness	5/10 (50.0)	–
*Strongyloides* infection status		
Hyperinfection	6/10 (60.0)	–
Dissemination	2/10 (20.0)	–
Not specified	2/10 (20.0)	–
*Strongyloides* testing		
Stool microscopy	4/10 (40.0)	–
Stool culture	1/10 (10.0)	–
Serology	4/10 (40.0)	–
Bronchoalveolar lavage	1/10 (10.0)	–
Sputum gram stain	1/10 (10.0)	–
Laboratory findings		
Leukocytosis, 10^3^/μL	6/10 (60.0)	15.6 (11.0–29.6)
Eosinophilia, %	6/10 (60.0)	0.54 (0.075–7.5)
COVID-19 treatment		
Prednisone equivalent ≥ 40 mg/day	7/10 (70.0)	–
Prednisone equivalent < 40 mg/day	1/10 (10.0)	–
Prolonged corticosteroid > 2 weeks	1/10 (10.0)	–
No corticosteroids	1/10 (10.0)	–
Biologics	3/10 (30.0)	–
*Strongyloides* treatment		
Ivermectin single dose	1/10 (10.0)	–
Ivermectin for 2 days	3/10 (30.0)	–
Ivermectin for 3–7 days	2/10 (20.0)	–
Ivermectin for > 7 days	1/10 (10.0)	–
Ivermectin for unknown duration	1/10 (10.0)	–
Albendazole along with ivermectin	2/10 (20.0)	–
ICU admission	5/10 (50.0)	–
Death	0 (0)	–

COVID-19 = coronavirus disease 2019; ICU = intensive care unit; IQR = interquartile range; WBC = white blood cell.

* Defined as the number of cases reported the variable divided by the number of the total cases.

## DISCUSSION

This is the first systematic review examining the association between COVID-19 and strongyloidiasis. Although the results showed limited evidence regarding the risk of strongyloidiasis among COVID-19 patients, there are cases of SHS in those who received corticosteroids or biologics for the treatment of COVID-19 for a limited duration, raising the need for increased awareness of the potentially critical condition.

The results of the two included observational studies suggest that the risk of strongyloidiasis in COVID-19 patients may be low, or patients could remain asymptomatic. However, the prevalence of strongyloidiasis during the COVID-19 pandemic could be higher than generally considered due to a potential underdiagnosis.^12^^,^^13^ Notably, the two observational studies were performed in Spain, where the overall seroprevalence of *S. stercoralis* was reportedly around 9%.^22^ Although there are several case reports from the country, most patients in the reports were migrants from endemic regions such as from Honduras or Bolivia, where the estimated prevalence of *S. stercoralis* is close to or more than 10%. Future general population-based surveillance plans may need to identify COVID-19 infection status, migrants, and countries of origin of patients with strongyloidiasis to avoid misinterpretation of endemicity.

Patients with strongyloidiasis and COVID-19 co-infection may have different clinical characteristics from those without COVID-19, as noted in the result that only 40% or less had a fever or respiratory symptoms despite the severity of strongyloidiasis. Typical clinical presentations of SHS and disseminated strongyloidiasis include extensive gastrointestinal symptoms concurrently noted with other organ system involvement, such as respiratory or dermatologic findings from the spread of larvae to other organs along with autoinfection. Because 90% had either severe or critical COVID-19 and required corticosteroid as treatment, SHS and disseminated strongyloidiasis, which are forms of severe manifestation of strongyloidiasis, could be precipitated by short-term immunosuppression with corticosteroids, which are a part of COVID-19 treatment. To examine if strongyloidiasis in COVID-19 is precipitated by immunosuppression or COVID-19 itself, further prospective studies are warranted to determine the causality. Interestingly, eosinophilia, a hallmark of parasitic infection, was not seen in 40% of the included cases, likely due to concurrent corticosteroid use. In 60% of the cases with eosinophilia, the extent of eosinophilia was primarily mild, with the median absolute eosinophil count of 0.54 × 10^3^/μL, which makes the diagnosis of strongyloidiasis challenging, leading to delay of the diagnosis. Detailed history taking along with physical examination including skin findings are crucial to aid the diagnosis.

Geography also seems to play a role in the prevalence of strongyloidiasis in COVID-19 patients. Among the included cases, 80% were born in tropical countries (defined as latitude between 23.43635° in the northern hemisphere and 23.43635° in the southern hemisphere), and 20% were born within a temperate climate zone, consisting of Italy and Spain. Interestingly, 37.5% of those in tropical countries were immigrants living either in Spain or the United States.^14^^,^^15^ Thus, detailed history taking, including travel and socioeconomic and immigration status, may be crucial in identifying those at risk.

Our study has several limitations. First, given the nature of the study, we are unable to elaborate on the causation of whether COVID-19 or corticosteroid use led to the development of strongyloidiasis or not. Second, due to a potential publication bias that more severe and complicated cases tend to be published, the cases included in the study may not be representative of the clinical characteristics of strongyloidiasis with COVID-19. Also, the results may not be generalizable because most articles were published from certain countries.

Despite the limitations, the study is the first to have extensively investigated the characteristics of strongyloidiasis in COVID-19, which might have been missed due to a lack of awareness. Given the potentially life-threatening and curable nature of strongyloidiasis, clinicians need to recognize that patients with strongyloidiasis in COVID-19 may present with atypical characteristics. Further research, in *Strongyloides*-endemic areas in particular, is warranted to clarify the prevalence of strongyloidiasis during the COVID-19 pandemic.

## Supplemental Materials


Supplemental materials
